# Microbubble Enhanced Echocardiography in Current Cardiology Practice

**DOI:** 10.31083/j.rcm2306202

**Published:** 2022-05-31

**Authors:** Mihai Strachinaru, Folkert J ten Cate

**Affiliations:** ^1^Department of Cardiology, Erasmus MC, 3000 CA Rotterdam, The Netherlands; ^2^Department of Biomedical Engineering, Erasmus MC, 3000 CA Rotterdam, The Netherlands

**Keywords:** microbubble enhanced echocardiography, ultrasound enhancing agents, contrast-enhanced ultrasound, contrast echocardiography, safety, review

## Abstract

Contrast-enhanced ultrasound imaging is a radiation-free clinical diagnostic tool that uses biocompatible contrast agents to enhance ultrasound signal, in order to improve image clarity and diagnostic performance. Ultrasound enhancing agents (UEA), which are usually gas microbubbles, are administered intravenously either by bolus injection or continuous infusion. UEA increase the accuracy and reliability of echocardiography, leading to changes in treatment, improving patient outcomes and lowering overall health care costs. In this review we describe: (1) the current clinical applications of ultrasound enhancing agents in echocardiography, with a brief review of the evidence underlying each of these applications; (2) emerging diagnostic and therapeutic applications of microbubble enhanced echocardiography (MEE), which rely either on the specific properties and composition of ultrasound enhancing agents or on the technical advances of clinical ultrasound systems; and (3) safety of MEE.

## 1. Introduction

**Fig. 1.**
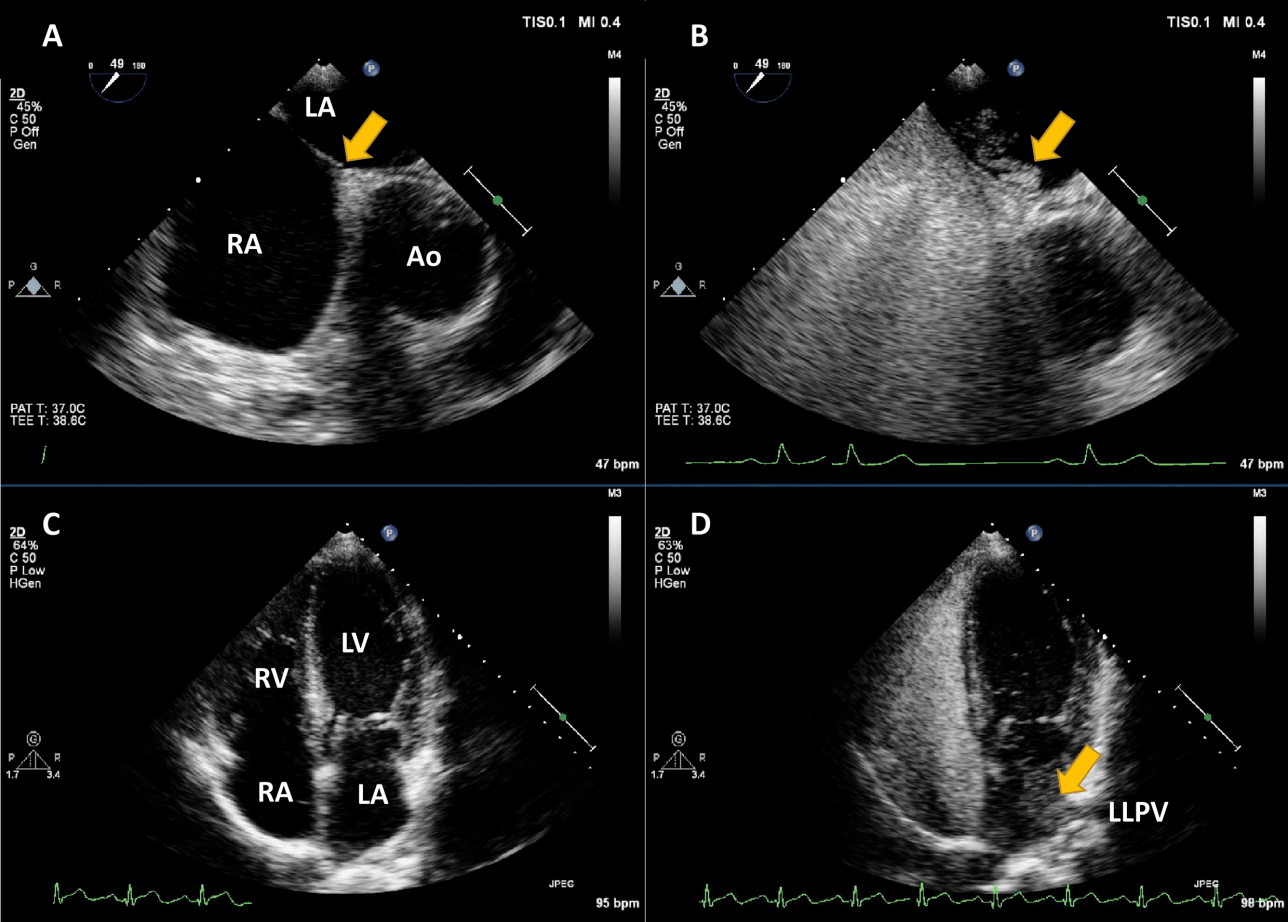
**Agitated saline injection for the detection of right-to-left shunts**. (A,B) Example of intracardiac right-to-left shunting through a patent Foramen Ovale; transoesophageal echocardiography in mid-oesophageal position at 45°. The right atrium (RA), left atrium (LA) and interatrial septum at the level of the fossa ovalis are seen (arrow). After the infusion of agitated saline in a peripheral vein, a thick cloud of bubbles is seen passing through the patent *foramen ovale* (PFO) (arrow). (C,D) Right-to-left shunting through an intrapulmonary shunt, detected with transthoracic echocardiography, apical 4-chambers view. All four cavities are visualised. After the infusion of agitated saline in a peripheral vein, the right cavities are completely opacified. Seven heart cycles after the appearance of contrast in the right heart, a thick cloud of bubbles is seen in the LA (arrow), coming from the left lower pulmonary vein (LLPV). Source: personal collection.

Echocardiography is the most commonly used imaging modality for assessing cardiac structure and function. However, an estimated 20% of echocardiographic studies may be suboptimal [1, 2]. Hand-agitated saline resulting in rapidly dissolving air bubbles have been used for decades to enhance the ultrasound reflection from the right heart. The bubbles resulting from agitated solutions are too short-lived and too large to pass through the pulmonary bed, a property which has been used for the selective detection of right-to-left shunting (Fig. 1, Video 1) [3]. Suboptimal delineation of the left heart structures stimulated the development of commercial ultrasound enhancing agents (UEA) [4, 5, 6], specifically engineered so that the bubbles would be small enough (<8 μm) to pass through the capillaries in the lungs. Microbubble enhanced echocardiography (MEE) is now a well-established method, with a clinical history of more than 30 years. The technical details covering contrast infusion, and the specific transmit-receive and radiofrequency processing algorithms specifically designed to detect UEA microbubbles have been described and summarized by numerous other works [7]. In this review we will focus on the use of stable UEA designed to transit through the pulmonary circulation in clinical echocardiography. We will address both current and future promising diagnostic and therapeutic applications.

**Video 1.** **Agitated saline injection for the detection of patent *foramen ovale***. (A,B) Transoesophageal echocardiography in mid-oesophageal position at 45°. The interatrial septum at the level of the fossa ovalis is seen. After the intravenous infusion of agitated saline, a thick cloud of bubbles is seen passing through the PFO. The movie corresponds to Fig. 1. The embedded movie may also be viewed at https://doi.org/10.31083/j.rcm2306202.

## 2. Methods

This narrative review considered landmark studies in the field of MEE, which were published between 1984 and 2021. Inclusion criteria were: studies with largely confirmed scientific impact, regardless of their size and design, which: (i) defined key technical and/or clinical aspects of the method; (ii) were very informative on specific clinical and/or technical applications; (iii) had been previously included in the European Association of Cardiovascular Imaging (EACVI) and American Society of Echocardiography (ASE) guidelines or (iv) were defined as landmark papers in the EACVI Key Reference Library (Contrast Echocardiography (https://www.escardio.org/), accessed on 15/03/2022). Only studies indexed in the PubMed database, and published in the English language were considered.

### Ethics Approval and Consent to Participate

All patients included in this review as clinical illustrations gave their informed consent for the anonymous use of their clinical data and echocardiographic images.

## 3. Ultrasound Enhancing Agents

The current generation of UEA consist of microbubbles of comparable size to red blood cells (less than 8 μm in diameter), consisting of a gas encapsulated in a shell. The properties of these bubbles in the ultrasound field, and hence the image generated, are conditioned by their size, type of shell and gas. The interactions of the bubbles with ultrasound are complex. Generally, these bubbles undergo compression and expansion when exposed to the ultrasound waves (Video 2). These interactions can be generally divided in stable cavitation (oscillation of the bubbles without destruction) or inertial cavitation (abrupt oscillation disrupting the shell). These oscillations produce scattering (i.e., linear signal), which is proportional with the bubble size. However, one of the most important properties of microbubble oscillation is that they produce non-linear signal [8], which can be detected by the ultrasound system at harmonic frequencies.

**Video 2.** **Ultrasound enhancing agent microbubble oscillation induced by an ultrasound pulse**. Images were obtained with a Brandaris-128 ultra-fast framing camera at a frame rate of 15.3 Mfps. (movie courtesy of Dr HJ Vos). The embedded movie may also be viewed at https://doi.org/10.31083/j.rcm2306202.

The microbubbles must be strong enough to resist significant destruction with the clinical ultrasound incident power output. This has been achieved by using lipid or albumin shells, encapsulating inert high-molecular weight gas cores [7, 9] (Table 1).

**Table 1.** **Current commercial ultrasound enhancing agents**.

Agent	Manufacturer	Shell	Gas	Average size (µm)
Optison	GE Healthcare	Albumin	Perfluoropropane	2–4.5
Definity/Lumify	Lantheus Medical Imaging	Lipid	Perfluoropropane	1.1–3.3
Sonovue/Lumason	Bracco Diagnostics	Amphiphilic phospholipid	Sulphur hexafluoride	2–3

## 4. Clinical Applications

### 4.1 Left Ventricular Opacification (LVO) 

**Fig. 2.**
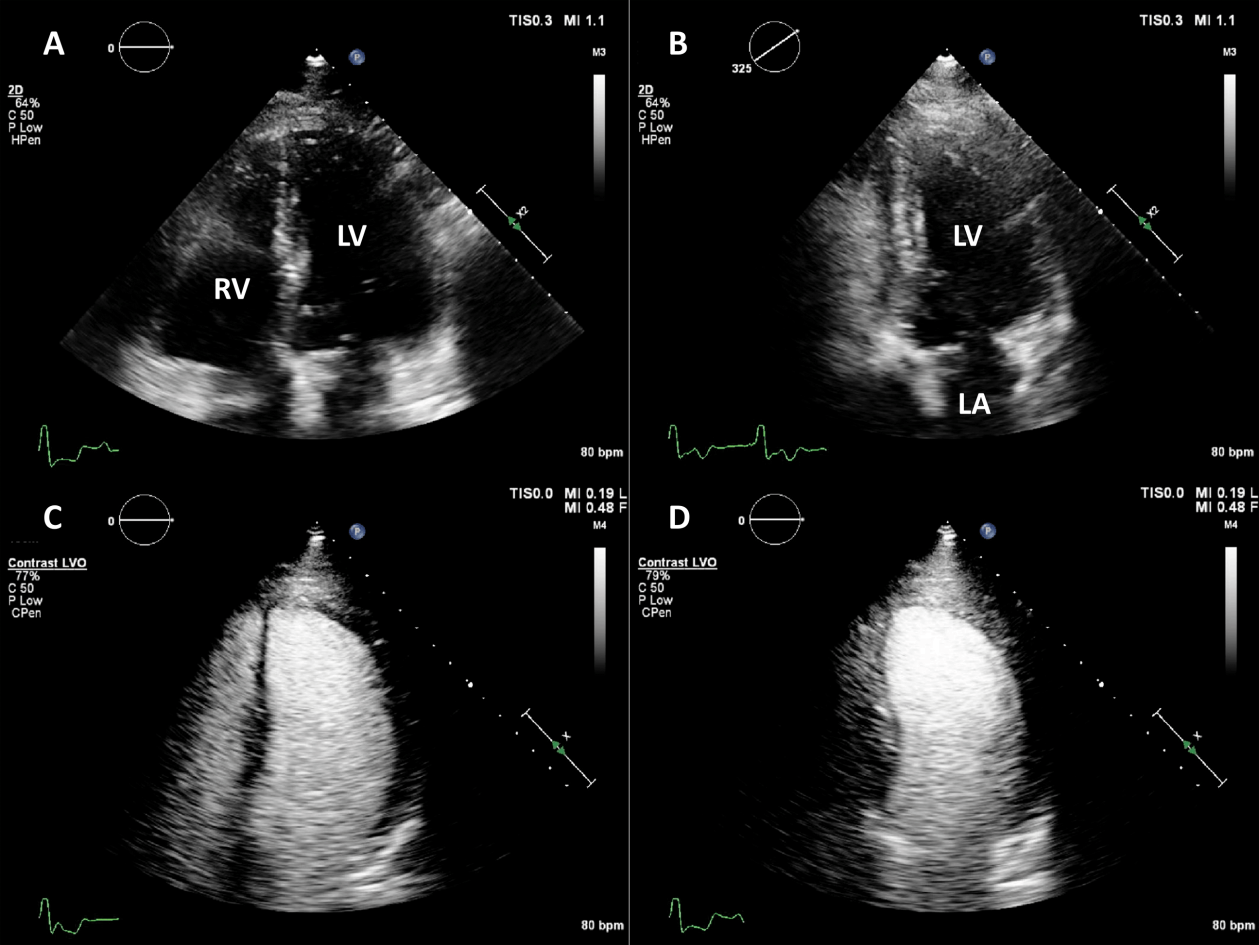
**Left ventricular opacification (LVO) for endocardial border delineation**. Example of baseline non-enhanced echocardiography images in apical 4-chambers (A) and apical 2-chambers (B) views, where the visualization of the endocardium is suboptimal over several segments. After intravenous injection of a bolus of UEA, there is full opacification of the LV cavity, with clear delineation of the endocardium in all segments (C, D). Source: personal collection.

The largest body of evidence for MEE concerns the indication for LVO for enhancing the endocardial borders [10, 11, 12, 13, 14, 15]. This is achieved by using repetitive intravenous boluses of UEA, and sometimes continuous low-dose infusion. Guidelines indicate the use of LVO to enhance the endocardial borders in cases when the LV dimensions, function or regional wall motion cannot be accurately assessed using non-enhanced ultrasound [7, 14]. The general “rule of thumb” is to use microbubble UEA in cases where two or more contiguous myocardial segments are not properly visualized with non-enhanced ultrasound [13]. Of course, recent years have seen tremendous improvement in image quality for clinical ultrasound systems. But despite the introduction of harmonic imaging as a standard, some images remain non-diagnostic (Fig. 2). Moreover, harmonic imaging represented a significant leap in MEE [16, 17], leading to the present-day contrast-specific imaging modalities.

#### 4.1.1 LV Size and Ejection Fraction (EF)

**Fig. 3.**
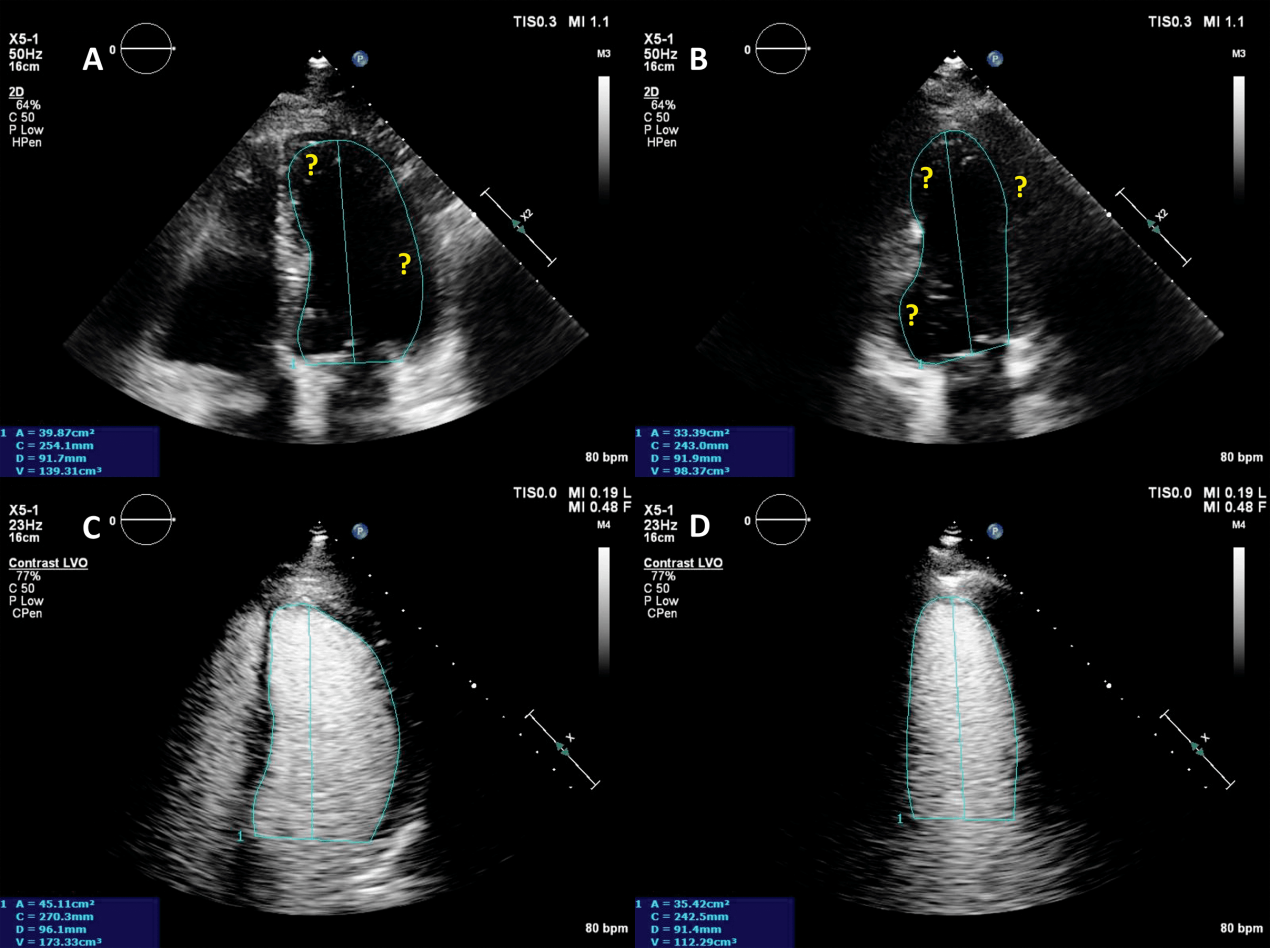
**Ejection fraction (EF) estimation**. (A,B) Non-enhanced ultrasound images. The endocardium is not clearly visible in several segments (question marks), making the volumes difficult to assess. (C,D) Contrast-enhanced images. The endocardial border is clearly defined, allowing for a biplane volume estimation. Moreover, the LV end-diastolic volumes in contrast-enhanced images is notably larger than the one on the non-enhanced images, probably because of a combination of insufficient image quality on native images, and exclusion of trabeculae and papillary muscles on contrast images. Source: personal collection.

By using UEA, enhanced echocardiographic measurements of LV volumes and ejection fraction are very close to the reference cardiac magnetic resonance (CMR) values [11, 12], and significantly less variable as compared to unenhanced imaging, even if baseline images are of good quality [18]. This significant difference in quality, information and accuracy leads to a clinical impact on diagnosis and management [6, 19, 20]. Echocardiographic estimates of LV volume tend to be larger when using LVO, mainly because it aids the exclusion of trabeculae (Fig. 3), making the measurements closer to their CMR counterparts [13].

3D MEE is not yet a standard. Although one multicenter study demonstrated that it may improve inter-observer variability and accuracy [11, 12], there are still limitations because of UEA destruction in the near field generated with 3D echo, which in other studies resulted in increased inter-observer variability [21].

As such, LV microbubble-enhanced echocardiographic volumes are not presented in the current guidelines for chamber quantification [22], and they should not be compared with the non-enhanced values mentioned in these guidelines. New reference ranges for LV enhanced echocardiographic volumes should be established by large studies.

The evidence regarding the use of MEE for linear size measurements of the LV is less strong. Recent monocentric studies suggest a benefit of measuring the interventricular septal size in hypertrophic cardiomyopathy with MEE, and the results may be closer to the reference CMR and smaller than non-enhanced values [23, 24]. However, precise reference ranges for MEE LV size and wall thickness in parasternal views are yet lacking, and CMR should be preferred whenever possible in the workup of HCM patients. Also, for the posterior wall thickness in the parasternal views MEE may surprisingly not be better than non-enhanced images [24], and this because the significant signal attenuation through interposition of the microbubbles in the right and left ventricular cavities. This is even more obvious at higher concentrations of UEA.

#### 4.1.2 Assessment of Regional Wall Motion at Rest

The significant improvement in endocardial delineation demonstrated a similarly significant benefit in the analysis of regional wall motion abnormalities [25]. When analyzed by a panel of experts, the accuracy in diagnosing wall motion abnormalities was highest for CMR (84%), followed by 2D MEE (78%) and 3D contrast (76%) [25]. In addition to this multicenter study, other works demonstrated the benefit of contrast imaging in the interpretation of LV wall motion in intensive care patients [26], or post myocardial infarction [19, 27]. The benefit becomes more obvious when used as point-of-care echocardiography, to rapidly identify wall motion abnormalities in patients with suspected coronary artery disease (Video 3).

**Video 3.** **Patients with non-specific thoracic pain and non-diagnostic electrocardiogram**. Non-enhanced images (left) in the parasternal short axis of the left ventricle at the level of the papillary muscles lack definition in the interventricular septum, no clear motion abnormality is seen. With contrast (right) the endocardium is clearly seen and hypokinesia is noted in the interventricular septum. The embedded movie may also be viewed at https://doi.org/10.31083/j.rcm2306202.

#### 4.1.3 Assessment of LV Wall Structure

Another application of LVO is morphological diagnosis, particularly in disease states which manifest in the artefact-prone LV apex. Beside possible foreshortening, the LV apex is prone to clutter and reverberation artefacts, while also having a weaker potential to generate harmonics because of its position in the near-field in apical views [28]. As such, apical forms of hypertrophic cardiomyopathy [29, 30], eosinophilic cardiomyopathy [31] and non-compaction cardiomyopathy [32] may escape detection with unenhanced ultrasound.

**Fig. 4.**
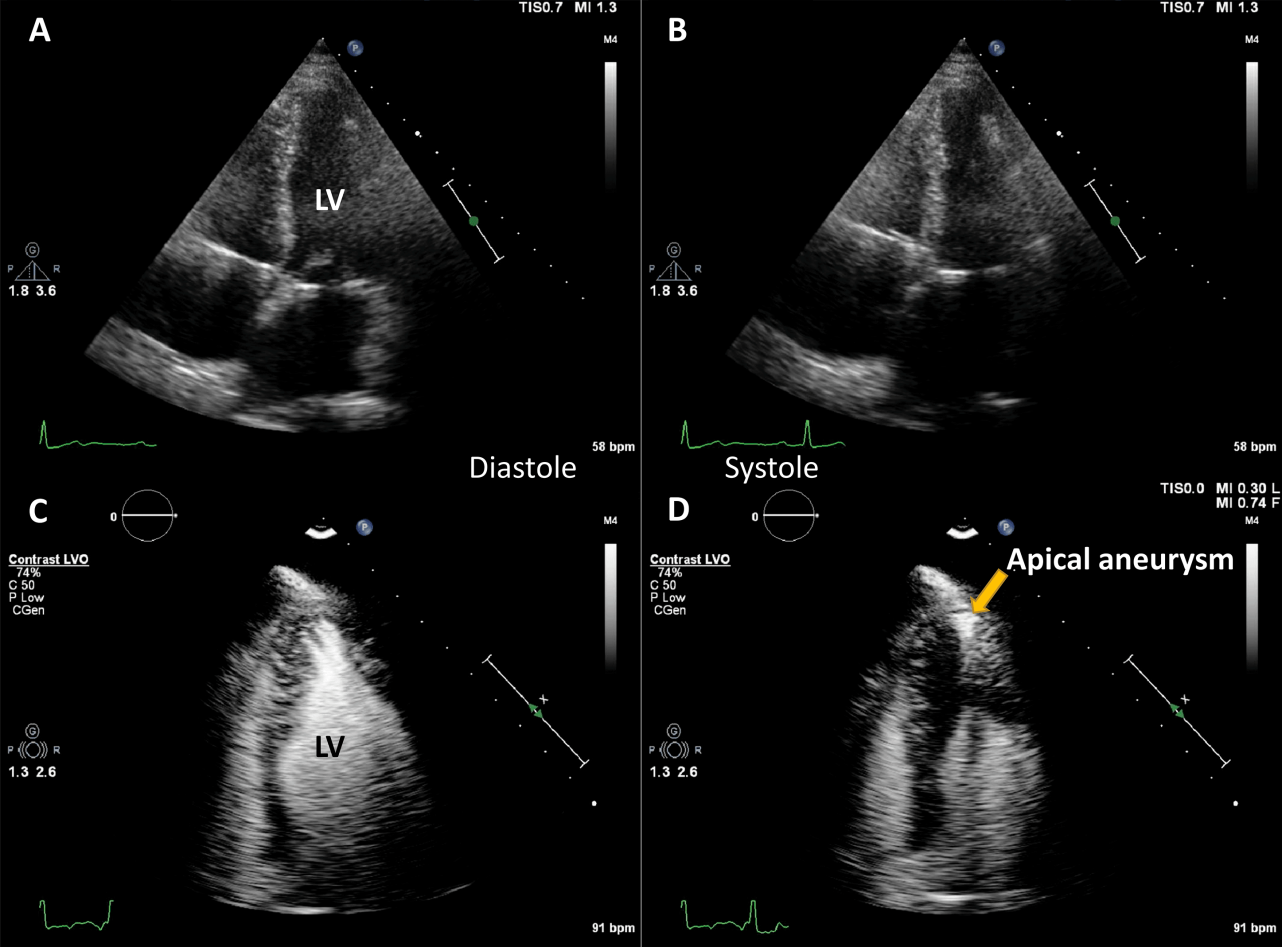
**Hypertrophic cardiomyopathy patient with very poor image in apical 4-chambers view**. (A,B) Native images, end-diastole (A) and end-systole (B). The endocardium of the lateral wall is not visible, and the apex cannot be seen. (C,D) Contrast-enhanced images, in the same moments in the cardiac cycle. The LV contour is clearly delineated, during systole there is complete cavity obliteration, with an apical aneurysm (arrow). Source: personal collection.

Numerous case reports and case series document the use of UEA in these instances [29, 30, 31, 32, 33]. Studies have also been performed demonstrating the added value of contrast-enhanced ultrasound in hypertrophic cardiomyopathy [34] (Fig. 4, Video 4).

**Video 4.** **Apical hypertrophic cardiomyopathy, microbubble-enhanced echocardiography**. The images correspond to the patient in Fig. 4. There is mid-cavity obliteration, with a dyskinetic apical pouch (apical aneurysm), which were not seen on native images. The embedded movie may also be viewed at https://doi.org/10.31083/j.rcm2306202.

#### 4.1.4 Left Atrial Appendage Visualization during Transoesophageal Echocardiography

**Fig. 5.**
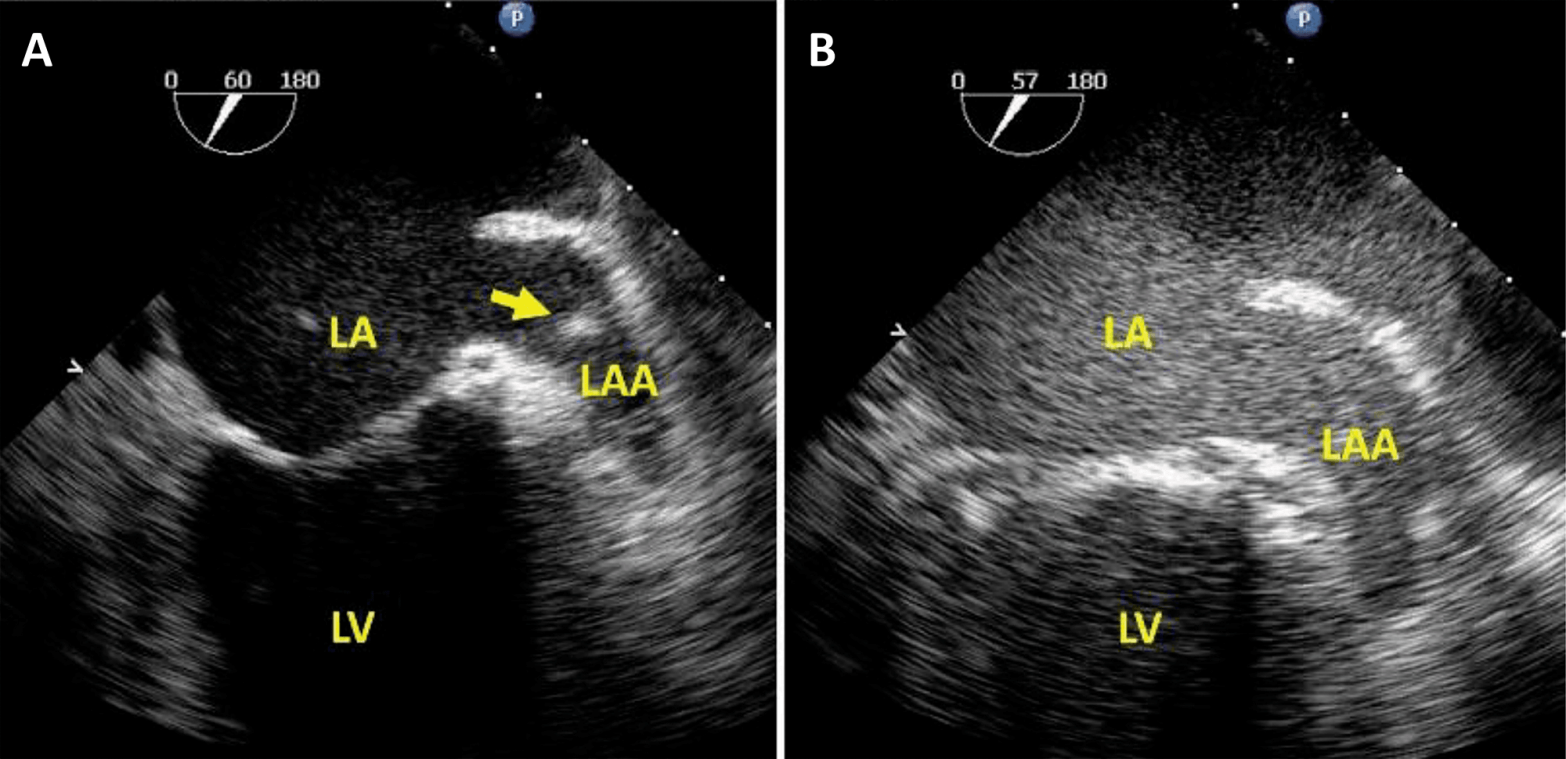
**Ultrasound-enhanced TEE used to facilitate the safety of an atrial fibrillation cardioversion procedure**. (A) Mid-esophageal view at ${\mathrm{60}}^{\circ }$, demonstrating a hyperechoic signal (arrow) inside the left atrial appendage (LAA), which could be an artefact, but a thrombus cannot be excluded because of “smoke” in the LAA. (B) By adding intravenous ultrasound enhancing agent, the LAA appears free of any abnormal echo. In the absence of thrombus, the cardioversion was successfully performed. Modified with permission from Doukky R *et al*. [36].

Transoesophageal echocardiography is an established method for assessing the left atrial appendage (LAA) for the presence of thrombi [35, 36] or to guide LAA interventions (Fig. 5, Ref. [36]). In case of LAA stasis, the dense spontaneous contrast may mask the presence of a small thrombus. Several studies demonstrated that the adjunction of UEA increases the diagnostic yield of the procedure [37, 38] and reduces subsequent strokes [39].

**Fig. 6.**
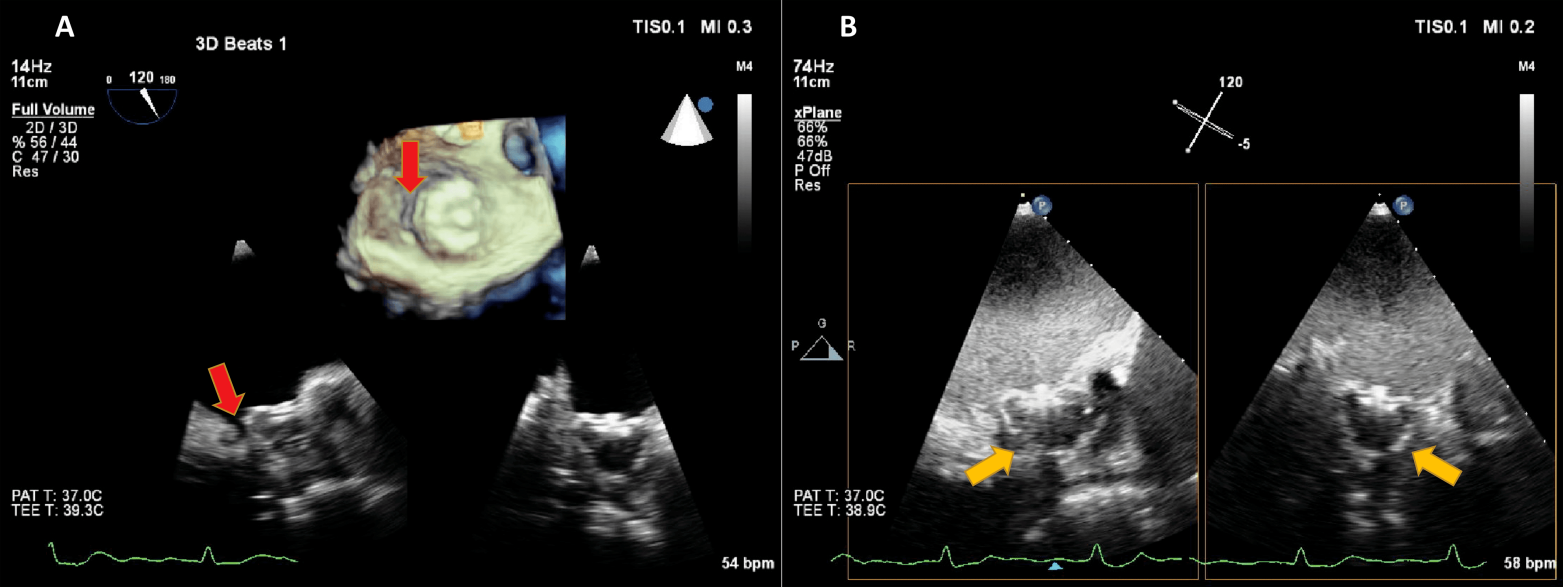
**Transoesophageal echocardiography in a patient with a Watchman left atrial appendage (LAA) closure device**. Microbubble-enhanced ultrasound was used in order to detect a suspected residual leak around the occluder. On non-enhanced multiplane and live 3D images (A) a gap is visible (red arrows) between the rim of the device and the LAA. Because of shadowing, the sealing of the LAA cannot be verified. By adding intravenous contrast (B) and using harmonic imaging with a mechanical index of 0.2, contrast is seen in the LAA, surrounding the device (yellow arrows), demonstrating incomplete sealing of the LAA. Source: personal collection.

However, contrast-specific transoesophageal applications are not available on all ultrasound clinical systems, therefore in such cases non-contrast mode harmonic 2D imaging may be used, with a mechanical index (MI) under 0.3 (Fig. 6, Video 5).

**Video 5.** **Microbubble-enhanced transoesophageal echocardiography biplane images focused on the left atrial appendage (LAA)**. In the LAA a closure device (Watchman) is present, but the sealing is incomplete, contrast can be seen all around the device, enhancing its borders. The patient corresponds to Fig. 6. The embedded movie may also be viewed at https://doi.org/10.31083/j.rcm2306202.

The significant improvement LVO provides in accuracy, reproducibility and confidence in the assessment of LV size, shape and function encourages the incorporation of this relatively low-cost method in the standard exploration of clinical patients. This has been recognized by the current guidelines [7, 13, 22]. It seems reasonable to systematically use UEA for LVO in patients in whom two or more segments are not adequately visualized with non-enhanced ultrasound.

### 4.2 Stress MEE (LVO)

**Fig. 7.**
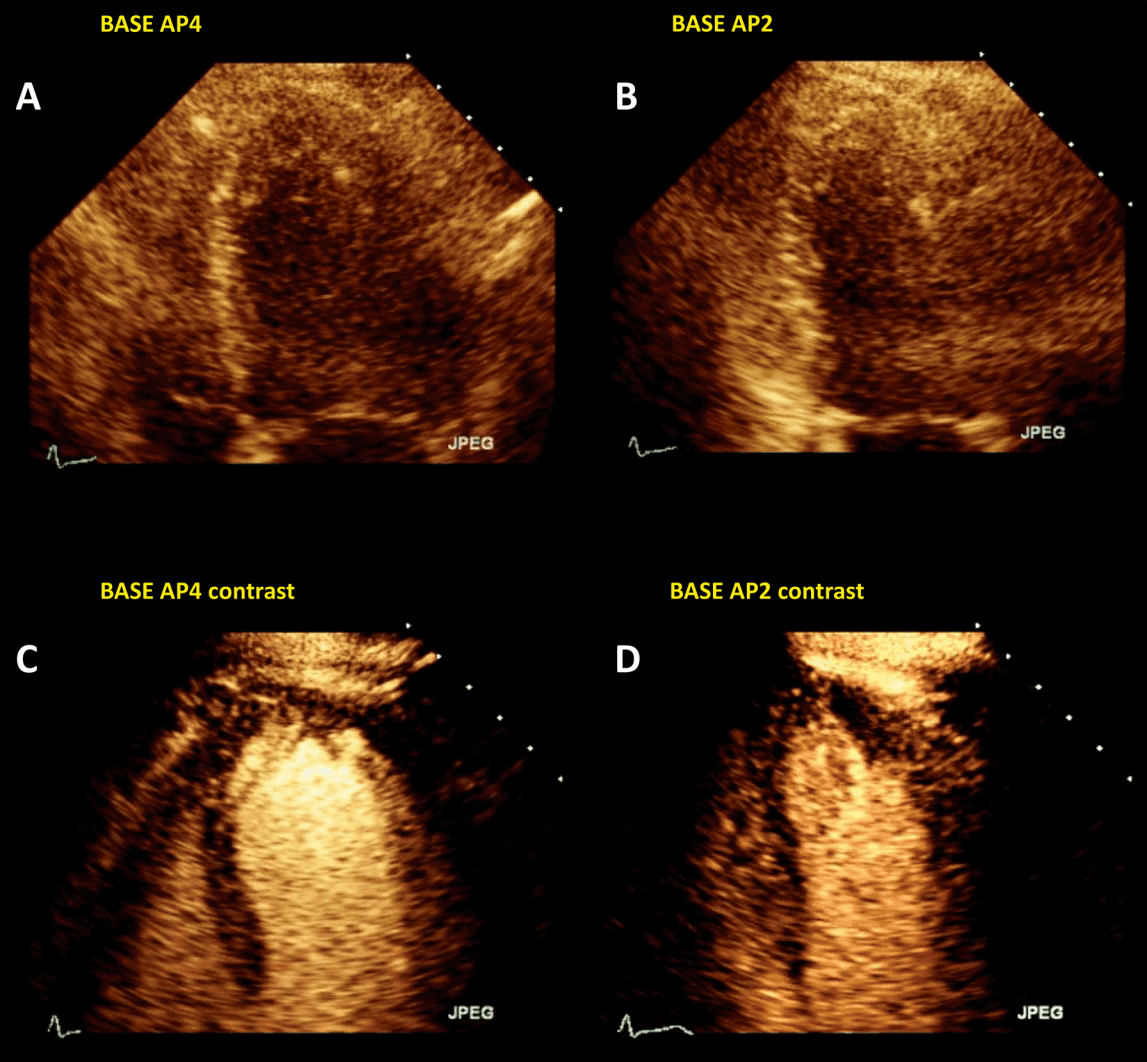
**Microbubble enhanced stress echocardiography**. Baseline non-enhanced images are recorded in apical 4 and 2 chambers (A,B), demonstrating insufficient delineation of the endocardial borders; With contrast (C,D) the LV contours become clearly visible. Source: personal collection.

The addition of UEA during stress echocardiography protocols is usually achieved through an LVO application with low-MI harmonic imaging (Fig. 7, Video 6). The result is an increase in the likelihood of a diagnostic test, a better visualization of all myocardial segments, study quality and reader confidence, as compared to invasive or non-invasive reference [40, 41, 42, 43]. The addition of UEA to non-enhanced studies resulted in a better agreement with coronary angiography, even in patients with intermediate coronary lesions [44]. Of course, the use of LVO in stress echo has the largest impact in patients with suboptimal image [45]. Nevertheless, contrast-enhanced ultrasound also improves the wall motion score and detection of regional wall motion abnormalities in patients with adequate image quality [46]. Contrast-enhanced dobutamine stress echocardiography provided adequate risk stratification in patients with increased cardiovascular risk due to obesity or suspected coronary artery disease [47, 48, 49].

**Video 6.** ** Dobutamine stress echocardiography, baseline images**. The upper panels are non-enhanced, LV walls and endocardial borders are difficult to see. The lower panels show the contrast-enhanced images, the LV contours and wall motion are clearly visible. The movie corresponds to Fig. 7. The embedded movie may also be viewed at https://doi.org/10.31083/j.rcm2306202.

In patients with incomplete visualization of at least 2 contiguous segments contrast should be used for stress echocardiography. In patients with adequate image quality, contrast could be used to assess the myocardial perfusion, in addition to wall motion [7, 13].

### 4.3 Myocardial Perfusion Imaging

#### 4.3.1 Detection of Coronary Artery Disease

**Fig. 8.**
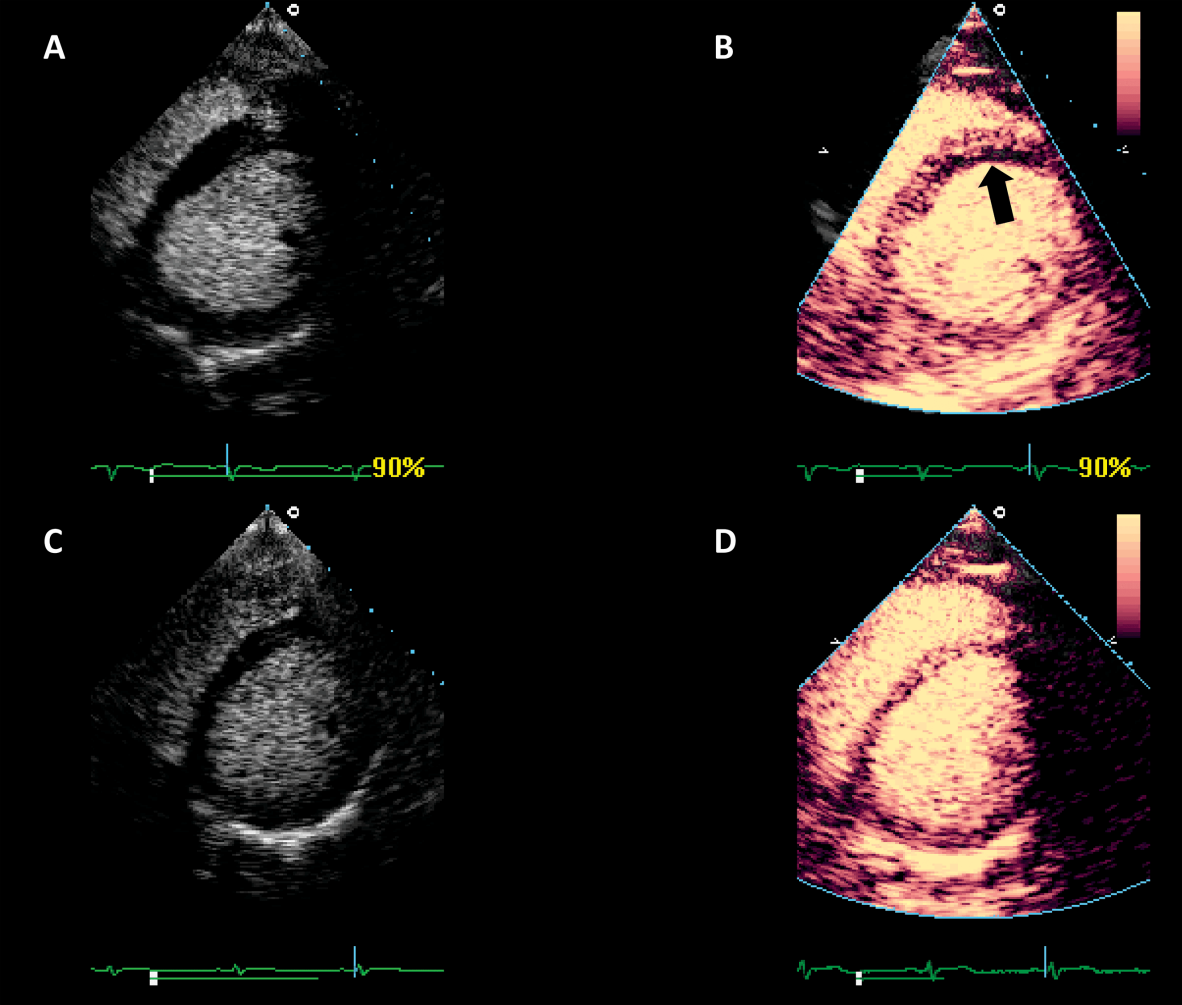
**Detection of coronary artery disease by using myocardial perfusion imaging**. Patient with dyspnoea but no typical chest pain. (A) LVO images demonstrate a discrete anomaly in the septal kinetics. (B) Myocardial perfusion imaging shows a subendocardial perfusion defect in the anterior and anteroseptal segments. Patient underwent coronary angiography and stent placing in the left anterior descendent coronary. (C) Follow-up study before discharge: LVO normal kinetics. (D) Follow-up perfusion study on discharge: homogeneous perfusion. Source: personal collection.

UEA have an intravascular behavior which mimics closely that of red blood cells [50], and an in vivo distribution to the intravascular compartment, making them ideal for assessing microvascular distribution in the tissue. The total blood volume in the coronary circulation at rest is around 12 mL/100 g of myocardial tissue [51] and 90% of this volume is found in the capillaries. If the myocardium is fully saturated during a continuous UEA infusion, the UEA in the myocardium will reflect the distribution of the capillary circulation [52]. This means that simply measuring the intensity of signal enhancement with UEA provides a quantitative measure of intact microcirculation in the myocardium. Using this approach, the extent and distribution of viable (perfused) myocardium can be assessed (Fig. 8, Video 7) with a sensitivity and specificity similar to single photon emission computed tomography (SPECT) techniques [41] or magnetic resonance imaging (MRI) [53].

**Video 7.** **Detection of coronary artery disease by using myocardial perfusion imaging**. Upper panels: acute phase; Lower panels: discharge. In the acute phase myocardial perfusion imaging (upper right panel) shows a subendocardial perfusion defect in the anterior and anteroseptal segments, with mild hypokinesia. Patient underwent coronary angiography and stent placing in the left anterior descendent coronary. Perfusion was homogeneous at discharge. The movie corresponds to Fig. 8. The embedded movie may also be viewed at https://doi.org/10.31083/j.rcm2306202.

Experimental data demonstrated that the myocardial capillary flow has a velocity of 1 mm/s within a beam elevation of 5 mm. This means that the total filling of the capillaries takes around 5 seconds (5 cardiac cycles at a heart rate of 60/min) at rest [54]. If for some reason the blood flow is slowed down (stenosis) or accelerated (vasodilation), this time will be modified accordingly [54]. During stress the myocardial blood flow increases 4–5-fold, which means that replenishment will be achieved in 1 to 2 seconds (2–3 cardiac cycles at a heart rate above 120/min).

**Fig. 9.**
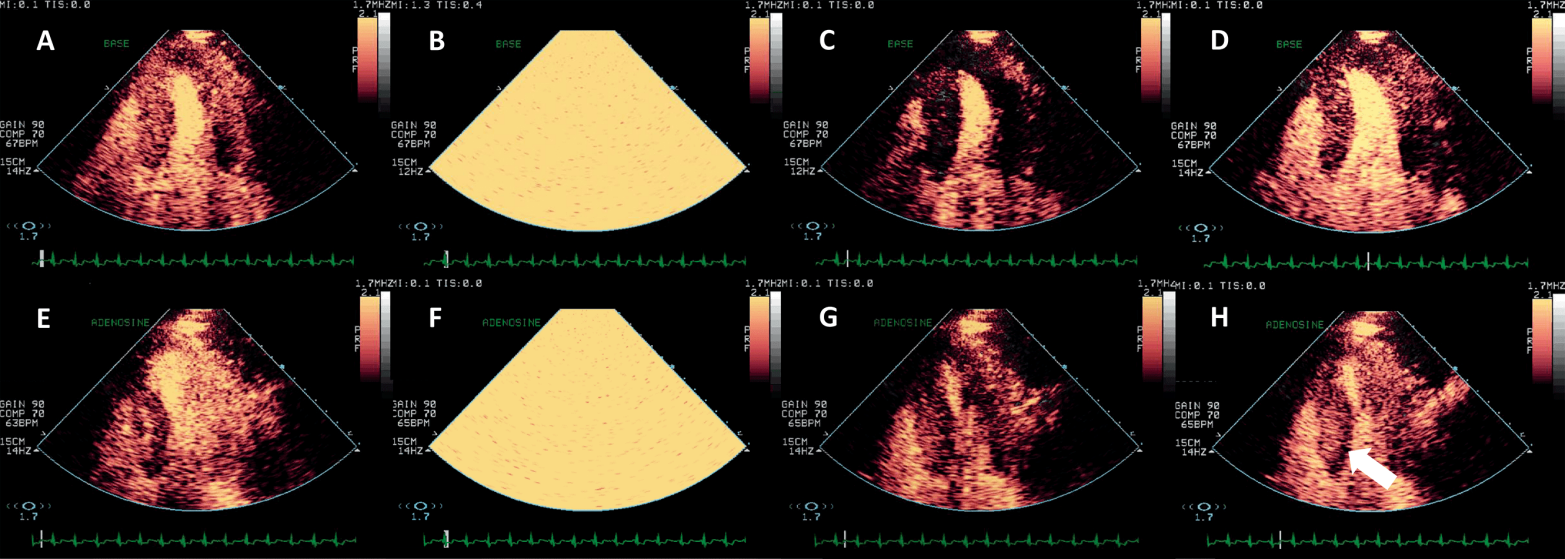
**Vasodilator stress echocardiography with Adenosine, using a low-MI setting and flash-replenishment technique**. (A–D) Baseline end-systolic frames before flash (A), flash (B), immediately post flash (C, myocardium is dark because of the destruction of contrast), and post-replenishment frame (D) when perfusion is again homogeneous. (E–H) After adenosine infusion, the same order of frames. (H) Post replenishment end-systolic frame demonstrating a subendocardial perfusion defect in the inferosetptal segements (arrow). Of note, during stress myocardial replenishment occurs faster than at rest because of the pharmacological vasodilation. The patient underwent coronary angiography and stent placing in the right coronary artery. Source: personal collection.

Assessing myocardial perfusion has incremental benefit over wall motion analysis in detecting coronary artery disease (CAD) [55]. The myocardial contrast signal obtained in a steady state (continuous infusion) can be normalized to the LV cavity signal and this represents the myocardial capillary volume [54]. By delivering a series of high-power (high mechanical index) ultrasound frames to the region of interest, cavitation and destruction of the UEA bubbles is initiated; the analysis of the progressive recovery of the contrast signal in the myocardium provides information on the myocardial capillary flow [54, 55, 56] (Fig. 9, Video 8).

**Video 8.** **Adenosine contrast-enhanced stress echocardiography, with a flash-replenishment cycle**. After the flash there is a persistent perfusion defect in the inferoseptal segments. The movie corresponds to Fig. 9. The embedded movie may also be viewed at https://doi.org/10.31083/j.rcm2306202.

The sensitivity and specificity of myocardial contrast stress echocardiography in detecting CAD are 83% and 79% respectively for a vasodilator stress (dipyridamole or adenosine) and 88% and 77% respectively for dobutamine or exercise stress studies [7, 57]. Two large multicenter studies demonstrated superior sensitivity of myocardial perfusion stress echocardiography as compared to SPECT, but lower specificity [58, 59]. The higher sensitivity may be due to the fact that SPECT only detects the myocardial blood volume, and not the kinetics of myocardial blood flow, as opposed to contrast-enhanced stress echocardiography, which can assess both [54]. The lower specificity may be related to artefacts during stress echocardiography, mainly in the apex (near-field destruction) and basal segments (far field attenuation in apical view).

There is now a large body of evidence supporting the added value of myocardial perfusion imaging over wall motion assessment alone in stress echocardiography [55, 60, 61, 62, 63, 64, 65, 66]. When using vasodilator stress, the use of high-power flash-replenishment technique is likely more important than during dobutamine or exercise stress, where wall motion abnormalities and perfusion defects may be more evident because of the higher oxygen demand during this type of stress [67].

#### 4.3.2 Myocardial Viability

As mentioned earlier, the intramyocardial UEA signal intensity correlates with the microvascular density in the area, and would naturally be lower in regions with high collagen content [68, 69]. Dobutamine stress echocardiography is routinely used for the assessment of myocardial viability. A contractile response during stress relates to the presence of the microvasculature and the presence of a blood flow reserve, both of which are particularly well predicted by MEE. An increasing body of evidence suggests that MEE is a useful and highly feasible technique for the evaluation of myocardial viability [53, 70, 71, 72].

#### 4.3.3 Coronary Flow Reserve

Quantitative approaches to evaluate myocardial perfusion or myocardial flow rate (the β-value = the rate at which the myocardial blood volume transits the tissue [54]) have been shown to correlate well with coronary flow, fractional flow reserve and positron emission tomography [41, 54, 73, 74, 75]. This can be achieved both with high as well as with low MI applications. For the high-MI the myocardium is first cleared of contrast (flash) and then replenishment is assessed with triggered high-MI imaging or continuous low-MI. The blood flow is estimated as the product of peak acoustical intensity (db) and flow velocity (db/s), and compared with the values obtained during stress, leading to a good correlation with invasive coronary flow reserve [73].

It is essential that in quantitative methods, relative indices are used, such as the ratio of stress and rest blood flow. The interaction between ultrasound power and microbubble concentration is variable in each patient and with each ultrasound system, which affects the absolute values of myocardial blood volume and blood flow velocity.

### 4.4 Intracardiac Masses

**Fig. 10.**
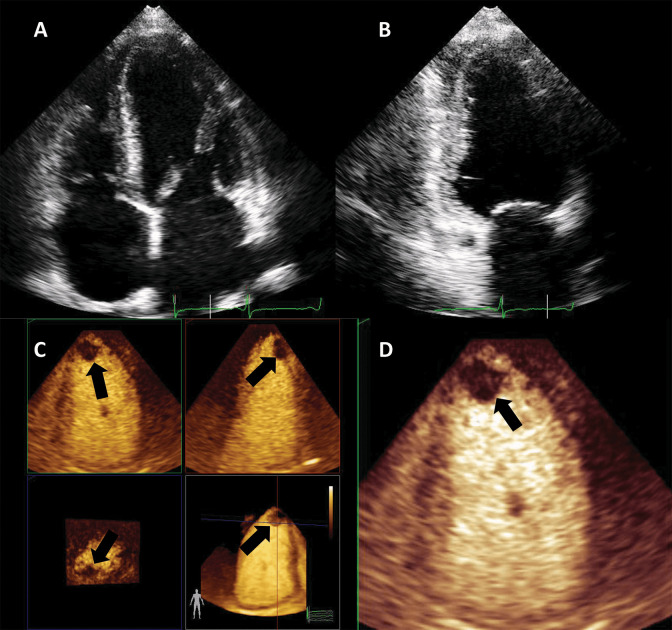
**Detection of an apical thrombus in a patient with severely depressed EF**. Native images in apical 4 (A) and 2 chambers (B) do not demonstrate the presence of an apical mass. (C) 3D contrast-enhanced echocardiography demonstrating an apical filling defect (thrombus-arrows). (D) Apical 2-chambers 2D contrast-enhanced image, the thrombus is present in the apical LV. Images modified with permission from Strachinaru *et al*. [79].

**Fig. 11.**
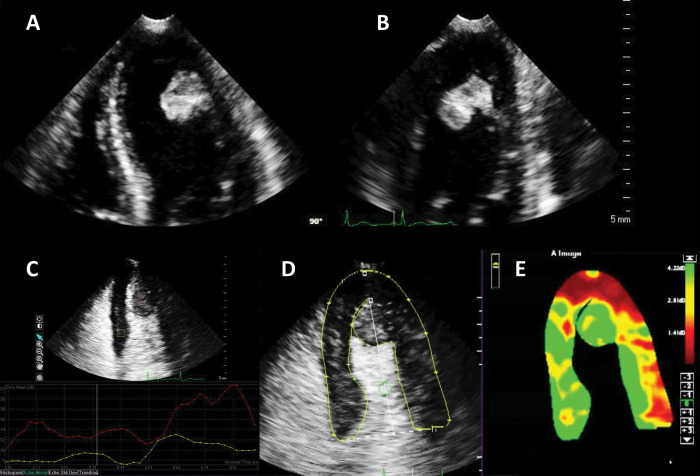
**Hyperechoic and hypermobile mass in a normal LV**. The image quality is good, and the mass is hyperechoic. (A) 4-chambers view. (B) 2-chambers view. (C) Signal intensity quantification after a flash-replenishment cycle. The replenishment of the mass is similar to the interventricular septum, but with higher intensity, signalling the presence of a capillary vascularisation, with a higher density than the myocardium. (D,E) Parametric map of signal intensity, demonstrating the same features of the mass. In this patient the mass was finally diagnosed as a hypervascular metastasis from a lung carcinoma. Images modified with permission from Strachinaru *et al*. [79].

UEA have largely been used in the detection of LV thrombi [76, 77, 78]. This is in fact an application of the LVO method, enhancing the delineation between the wall, cavity and the thrombus mass (Fig. 10, Ref. [79]). However, prognostic implications for small mural thrombi are not clear. Myocardial perfusion detection, as described above, may help to diagnose small intracardiac or even intramural masses, by the presence and the dynamics of the vascularization inside the mass [80]. By adding a quantitative approach, it may even be possible to differentiate not only thrombi from tumors, but also benign from malignant tumors [79] (Fig. 11, Ref. [79]).

### 4.5 Cardiac Contrast-Enhanced Ultrasound in Children and Adolescents

In children, image quality in transthoracic echocardiography is usually considered to be significantly better than in adults, due to body size, depth of the region of interest and degree of soft tissue hydration. This allows for the use of high-resolution high-frequency transducers, and also implies that the use of ultrasound enhancing agents is less frequent. However, echocardiographic images are not always diagnostic in this patient population, and other imaging modalities also tend to be used less than in adults (cardiac computed tomography being a radiation imaging technology, and cardiac magnetic resonance often requiring sedation for smaller children). All the clinical applications described above (LVO, myocardial contrast, intracardiac mass detection) have potential indications in pediatric patients [81, 82]. MEE has been described in the detection of coronary involvement in Kawasaki disease or in congenital heart disease [81, 83, 84]. In complex congenital heart disease, the acoustic window may be limited by post-surgical anatomy and the interposition of strong reflectors (implanted material). In these patients however, there is a clear benefit from the precise delineation and measurement of the right and left ventricle, which may have unusual shapes [82].

The safety of UEA in children has been a subject for debate [13, 85, 86, 87]. In 2016 the FDA removed the intracardiac shunt contraindication from all UEA labels. There are remaining concerns, in particular in congenital patients with large right-to-left shunting, that the UEA microbubbles may directly enter the arterial circulation, potentially inducing microvascular obstruction [13]. There are no current studies on the safety of UEA in patients under the age of 5.

For these reasons, the use of contrast agents is currently considered safe only in pediatric and congenital heart disease patients older than 5, without large right-to-left shunts. Further studies are needed in this direction, and a thorough benefit/risk assessment should be performed in each case. Optimal dosing of UEA in children corresponds to body weight.

### 4.6 Emerging Applications

#### 4.6.1 Intracardiac Flow Tracking and Quantification

**Fig. 12.**
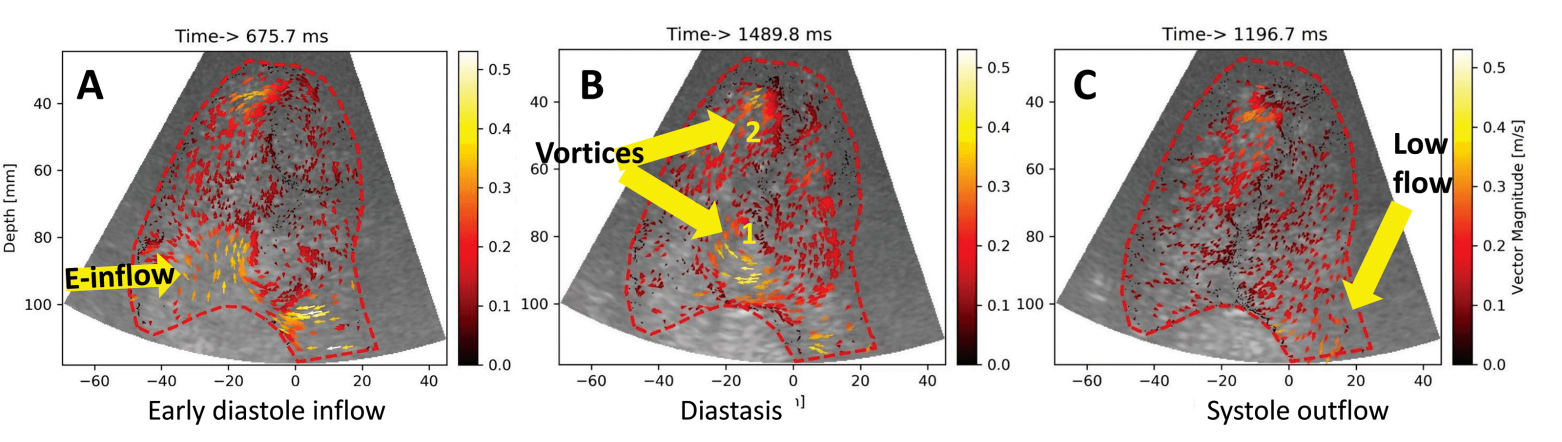
**High frame rate echo particle velocimetry (HFR echoPIV) in a heart failure patient**. (A) early diastolic inflow, corresponding to the Doppler E wave; (B) intraventricular flow in mid-diastole, during diastasis; (C) Flow during mid-systole. Inflow, outflow direction and magnitude can be visualized and quantified. Rotational flow (vortices) can also be seen and measured. Source: personal collection.

Blood flow in the heart is classically imaged using colour Doppler, which has some inherent disadvantages: angle-dependency, relatively low frame rate, low velocity range and semi-quantitative nature. New ultrasound techniques, generally referred to as vector flow imaging, can estimate the location, direction and magnitude of the velocity vectors that describe flow in a region of interest. One such technique, Echo-Particle Image Velocimetry (echoPIV), tracks the speckle of ultrasound contrast agent (UEA) microbubbles [88, 89]. These intricate echoPIV flow fields may offer additional meaningful insights, such as derived quantities (vorticity, circulation, kinetic energy, kinetic energy dissipation) [90]. The precise clinical meaning of these derived parameters still needs to be investigated. One of the limitations of conventional echoPIV is the relatively low frame rates permitted by conventional line-scanning based ultrasound imaging (maximum ~100 Hz). In contrast, high frame rate (HFR) echoPIV, using diverging-wave transmit sequences, allows for frame rates in the kHz range and makes tracking of fast flow in the left ventricle (LV) possible [90, 91, 92, 93] (Fig. 12, Video 9). However, these methods are still in development as clinical high frame rate imaging is still far from implementation [94].

**Video 9.** **High frame rate echoPIV in a heart failure patient**. Individual bubbles are tracked in their motion, and their direction and velocity represented with arrows. This is both a qualitative and quantitative method, allowing to estimate velocity anywhere in the region of interest (here the LV end-diastolic contour, traced with a red dotted line). The movie corresponds to Fig. 12. The embedded movie may also be viewed at https://doi.org/10.31083/j.rcm2306202.

#### 4.6.2 Ultrasound Targeted Microbubble Destruction (UTMD) and Derived Applications

**Fig. 13.**
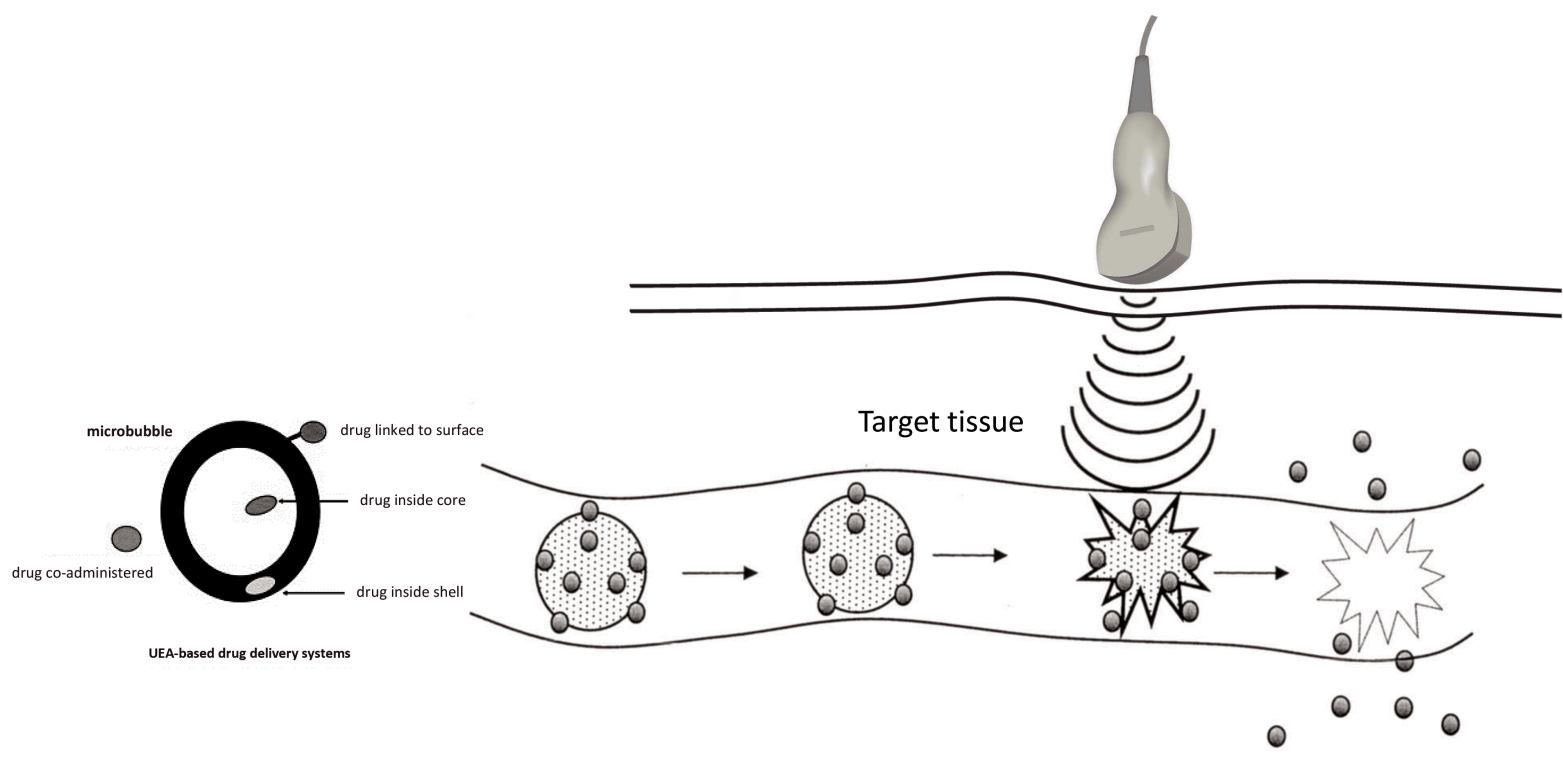
** Theoretical principle of ultrasound targeted microbubble destruction (UTMD) applications**. The bubbles can be “fitted” with a drug/gene/other marker, which can be released at the target site by destroying the bubbles with high-MI pulses. Source: personal collection.

Recent studies demonstrated that targeted drug and gene delivery can be done non-invasively. Genes or other substances may be incorporated on the surface of custom-generated microbubbles, or even inside the shell, and then destroyedas they reach the target area by using high-energy ultrasound pulses [95, 96] (Fig. 13). Because the UEA microbubbles are purely intravascular, delivery occurs to the vascular endothelium, but the ultrasound field and the energy generated by cavitation of the microbubbles can facilitate transfection into the extravascular tissue also [97, 98, 99]. Ongoing work is directed towards improving transfection of the drugs/genes into the target cells.

High-MI ultrasound pulses induce cavitation, which can increase local blood flow through the generation of nitric oxide [100]. This particular effect of UTMD has been recently used to improve perfusion in sickle-cell anemia [101], and in the myocardium in animal models [102].

#### 4.6.3 Sonothrombolysis

High-MI ultrasound pulses have the potential to disrupt and dissolve intravascular thrombi [103, 104, 105, 106]. Preliminary studies have demonstrated the recanalization of thrombosed vessels, without the adjunction of any drug treatment [103]. Preliminary clinical studies in ST-elevation myocardial infarction (STEMI) patients showed that guided high-MI pulses improve early recanalization rates and restore microvascular flow, reducing the infarct size [106]. Future and ongoing studies focus on the use of sonothrombolysis in acute coronary syndromes and stroke.

#### 4.6.4 Molecular Imaging

UEA are distributed strictly intravascular. By attaching certain ligands on their surface, they can target the surface of dysfunctional endothelium in certain diseased areas. This is achieved through modified UEA microbubbles, which are relatively easy to engineer and in large quantities. By using contrast-specific imaging modalities, and imaging after the normal time of clearance of the free contrast microbubbles, the diseased area can be selectively highlighted [107]. This method has been used in myocardial ischemia, allograft rejection, myocarditis and angiogenesis [108, 109, 110, 111].

All these cutting-edge applications of MEE in the diagnosis and treatment of heart disease are mostly at the stage of early clinical translation, but they have shown clinical promise and are gaining momentum through the innovative work of several research groups.

### 4.7 Safety

Following their initial clinical use in the 90’s, ultrasound enhancing agents underwent a period of limited use, induced by fears of adverse events [112]. However, subsequent large studies demonstrated that in various settings (inpatients, outpatients, critically ill, rest or stress testing), no excess in mortality or myocardial infarction was observed when compared to control populations [113, 114]. In critically ill patients on mechanical circulatory support devices data has only been reported in a few small single-center reports [20, 115, 116]; however, none of these studies noted supplementary adverse events in this patient population. Furthermore, contrast-enhanced stress echocardiography was not associated with an increase in adverse events [117, 118, 119].

Rare (between 1/1000 and 1/10000 patients) side effects have been noted with contrast agents, usually mild and transient (headache, nausea, dizziness, paraesthesia, taste disturbances or reactions at the injection site). Serious allergic reactions have a very low incidence [120] (considered low-risk, with an incidence of 0.005–0.015%).

Therefore, the clinical use of MEE is considered very safe in all its applications. Meanwhile the FDA has lifted the contraindications initially issued in the 2007 “black box warning” [112]. The only absolute contraindications persisting today are in patients with known or suspected large intracardiac shunting and those with hypersensitivity to the UEA.

Intracoronary administration is also considered contraindicated, despite its systematic and uneventful use in hypertrophic cardiomyopathy patients undergoing septal ablation.

It is recommended that all personnel in contact with a patient during any MEE study should be familiar with early identification of an allergic reaction and the appropriate treatment. Allergy kits including auto-injectable epinephrine should be available and easily accessible [7, 13].

## 5. Conclusions

Contrast-enhanced echocardiography is a mature technique, with an established safety profile allowing for its routine clinical use. Yet, in spite of extensive clinical experience and research, contrast-enhanced echocardiography remains underused, largely due to insufficient experience of the clinicians and unjustified fear of adverse effects. Through this review we covered the current and future perspectives of MEE, which we hope will facilitate the understanding and incorporation of this method in everyday clinical practice.
